# Exosomal ssc-miR-1343 targets FAM131C to regulate porcine epidemic diarrhea virus infection in pigs

**DOI:** 10.1186/s13567-024-01345-3

**Published:** 2024-07-22

**Authors:** Weiyun Qin, Jing Jiang, Jiayun Wu, Yunxiao Xie, Zhengchang Wu, Mingan Sun, Wenbin Bao

**Affiliations:** 1 College of Animal Science and Technology, Yangzhou University, Yangzhou, 225009 China; 2 Key Laboratory of Applied Technology on Green-Eco-Healthy Animal Husbandry of Zhejiang Province, College of Animal Science and Technology, College of Veterinary Medicine, Zhejiang A&F University, Hangzhou, 310000 China; 3 Institute of Comparative Medicine, College of Veterinary Medicine, Yangzhou University, Yangzhou, 225009 China; 4 Jiangsu Agri-Animal Husbandry Vocational College, Taizhou, 22530 China

**Keywords:** Pigs, porcine epidemic diarrhea virus, exosomes, Ssc-miR-1343, FAM131C, immune response

## Abstract

The porcine epidemic diarrhea virus (PEDV) causes diarrhea in piglets, thereby causing very significant economic losses for the global swine industry. In previous studies, it has been confirmed that microRNAs (miRNAs) play an important role in the infection caused by PEDV. However, the precise molecular mechanism of miRNAs in the regulation of PEDV infection is still not fully understood. In the present study, we utilized miRNA-seq analysis to identify ssc-miR-1343 with differential expression between PEDV-infected and normal piglets. The expression of ssc-miR-1343 was detected in isolated exosomes, and it was found to be significantly higher than that in the controls following PEDV infection. The ssc-miR-1343 mimic was found to decrease PEDV replication, whereas the ssc-miR-1343 inhibitor was observed to increase PEDV replication, and ssc-miR-1343 was delivered by exosomes during PEDV infection. Mechanistically, ssc-miR-1343 binds to the 3′UTR region of FAM131C, down-regulating its expression, and FAM131C has been shown to enhance PEDV replication through simultaneously suppressing pathways associated with innate immunity. The ssc-miR-1343/FAM131C axis was found to upregulate the host immune response against PEDV infection. In conclusion, our findings indicate that the transport of ssc-miR-1343 in exosomes is involved in PEDV infection. This discovery presents a new potential target for the development of drugs to treat PEDV.

**Supplementary Information:**

The online version contains supplementary material available at 10.1186/s13567-024-01345-3.

## Introduction

Porcine epidemic diarrhea (PED) is an acute, highly contagious intestinal disease caused by the porcine epidemic diarrhea virus (PEDV). The virus affects pigs of all ages and is characterized by symptoms such as vomiting, watery diarrhea, and dehydration. PEDV primarily infects the small intestinal epithelial cells in vivo, causing significant morbidity and mortality in piglets. The mortality rates in piglets under 7 days of age can exceed 80% [1]. Outbreaks of PED in East Asia (since 2010) and North America (since 2013) have reinvigorated the ongoing research on porcine coronaviruses, which were initially discovered in 1978 [2].

PEDV is classified under the genus Alphacoronavirus within the Coronavirus family. Its particles display a polymorphic nature, characterized by the presence of a corona-like protein enveloping the outer capsule membrane. Additionally, the membrane exhibits fibrils with an approximate length of 10–13 nm. PEDV has a length of approximately 28 kb (excluding the poly A-tail) and contains the following four structural proteins: spike (S) protein, envelope (E) protein, membrane (M) protein, and nucleocapsid (N) protein, as well as nonstructural proteins [3].

Since the extensive spread of PED, vaccination has been widely implemented as a primary preventative measure against PEDV infection in pigs. However, since 2010, the effectiveness of vaccination has been significantly compromised due to the emergence of highly mutated strains of PEDV. Consequently, the available vaccines are not fully effective in reducing the incidence and lethality of PEDV in piglets [4]. The pursuit of novel strategies for combatting PEDV has emerged as a pressing requirement for the sustainable growth of the swine industry.

MicroRNAs (miRNAs) are a class of small noncoding RNAs that function in post-transcriptional regulation of gene expression [5], and are important regulators of host gene expression in viral infections [6]. MiRNAs have been found to have a close association with viral infection and replication. Moreover, there is a growing body of evidence indicating that host miRNAs can bind to various RNA viruses and effectively regulate their pathogenesis. In the context of host–virus interactions, miRNAs have been identified as key regulators that directly target the viral genomic RNA to inhibit viral replication [7].

Although some studies have investigated the microRNA expression profile during PEDV infection, the majority of these miRNA-seq studies have primarily focused on the cellular level, leaving a gap in our understanding of the organism-level landscape [8–10]. Furthermore, there is a lack of research exploring the mechanisms that underly PEDV infection. Zheng et al. [11] demonstrated that microRNA-221-5p exerted inhibitory effects on the replication of PEDV through the targeting of the genomic viral RNA and activation of the NF-κB pathway. Zhao et al. [12] demonstrated that exosomal miRNA‑328‑3p specifically targeted ZO‑3 and effectively suppressed the proliferation of PEDV. MiR-615 has been identified as playing a facilitating role in the replication of the PEDV through targeting of IRAK1, thereby inhibiting the expression of type III interferon [13]. In our previous study, it was discovered that miR-129a-3p effectively suppressed the replication of PEDV through the specific targeting of the EDA-mediated NF-κB pathway in IPEC-J2 cells [14].

Exosomes are small, single-membrane, secreted organelles of ~30 to ∼200 nm in diameter that have the same topology as the cell membrane and are enriched in selected proteins, lipids, nucleic acids, and glycoconjugates [15]. Exosomes affect the pathophysiological processes of the body in various diseases, and are similar to viruses in biogenesis, a process widely involved in many viruses’ replication, transmission, and infection. Virus-associated exosomes can simultaneously promote immune escape and activate the antiviral immune response of the body, which bidirectionally modulates the immune response [16]; meanwhile, exosomes are also enriched with small molecules such as the miRNAs. Therefore, investigating the impact of miRNAs in exosomes on the process of PEDV infection represents a significant topic for research.

Collectively, there are numerous porcine miRNAs whose functions and mechanisms during PEDV infection remain to be elucidated. In this study, we utilized samples that were previously collected by our research team, consisting of PEDV-infected and normal samples, for miRNA-seq analysis. The objective of this analysis was to identify differentially expressed miRNAs (DE miRNAs) in the jejunal mucosa of piglets. Furthermore, we identified miR-1343, a differential expression miRNA, which is localized in exosomes. Therefore, we aimed to investigate the functional role and underlying mechanisms of ssc-miR-1343, a miRNA with potential antiviral properties, found in exosomes. In addition, we further enhanced the understanding of miRNAs in the mechanism of host resistance to PEDV infection.

## Materials and methods

### Animals, cell lines, virus, and plasmids

PEDV-infected and normal piglets, which were obtained and described in our previous study [17], were utilized as the same samples for performing the miRNA-seq. In brief, 28 infected 8-day-old piglets with clinicopathological features of porcine epidemic diarrhea (vomiting, dehydration, and watery diarrhea) were chosen from a pig farm in Jiangsu Province. Simultaneously, 15 healthy 8-day-old piglets were selected from the same feeding environment. In the diarrheic pigs, only PEDV was detected through pathogen testing, while all of the control pigs tested negative for any pathogens. IPEC-J2 cells, Vero cells, and 293 T cells were cultured in Dulbecco’s Modified Eagle Medium (DMEM; Thermo Fisher Scientific) supplemented with 10% fetal bovine serum (Gibco). The PEDV CV777 strain was cultured in Vero cells following the previously described protocol [17]. Regarding the PEDV infection in IPEC-J2 cells, the cells were subjected to PEDV infection at a multiplicity of infection (MOI) of 0.1. The PEDV inoculum was removed at 2 h post-infection (hpi), and the cells were subsequently collected at 24 hpi for further experimentation. The ssc-miR-1343 mimics and inhibitor oligos were synthesized at the Shanghai GenePharma Co., Ltd (Shanghai). The pGL3-basic vectors were utilized to generate FAM131C-WT1, FAM131C-MUT1, FAM131C-WT2, and FAM131C-MUT2 plasmids. The pCDNA3.1 (+) vectors were utilized for the construction of plasmids overexpressing FAM131C. All transfections were conducted using jetPRIME (Polyplus) in accordance with the prescribed protocol.

### Data analysis of miRNA-seq and RNA-seq

RNA isolation was performed with the TRIzol method. For miRNA-seq analysis, 3 μg of total RNA per sample was utilized as the input material for constructing the small RNA library. The sequencing libraries were generated using the NEBNext^®^ multiplex small RNA library prep set for Illumina^®^ (NEB). The clustering of the index-coded samples was conducted on a cBot cluster generation system utilizing the truSeq SR cluster kit v3-cBot-HS (Illumina). After the generation of clusters, the library preparations underwent sequencing on an Illumina Hiseq 2500/2000 platform, resulting in the generation of 50 bp single-end reads. The threshold for selecting the differentially expressed genes was established as a *p*-value adjusted for multiple testing (padj) value < 0.01 and | log_2_(foldchange)|> 1. In order to perform RNA-seq analysis, duplicate samples were collected from both the FAM131C overexpression and control IPEC-J2 cells. These libraries were generated using the NEBNext^®^ Ultra™ RNA Library Prep Kit for Illumina® (NEB). The AMPure XP system (Beckman Coulter) was employed for the purification of fragments with a length ranging from 250 to 300 bp. Then, the PCR process was conducted. The Agilent Bioanalyzer 2100 system, manufactured by Agilent Technologies, was employed for the purification of the PCR products. RNA-seq analysis was conducted using an Illumina HiSeq platform following established protocols. The threshold for statistical significance was established as a padj value < 0.05.

### Real-time quantitative PCR

The extraction of total RNA was performed using the TRIzol method. Complementary DNA (cDNA) synthesis was performed using the PrimeScript RT reagent kit (TaKaRa). Real-time quantitative PCR (RT-qPCR) was conducted using the qPCR SYBR Green Master Mix (Vazyme). For the analysis of miRNA, the cDNA synthesis and RT-qPCR reactions were conducted using the miRNA 1st strand cDNA synthesis kit and the miRNA universal SYBR qPCR master mix, respectively. GAPDH and U6 were employed as the reference genes for RT-qPCR. The details of the primers are provided in Additional file 1. The relative quantification results were determined employing the comparative Ct (2^−ΔΔCt^) method.

### Exosome extraction and identification

A volume of 10 mL of the supernatant from IPEC-J2 cells was initially collected. The collected sample was then subjected to centrifugation at 3000 × *g* for 10 min at 4 °C in order to eliminate the cells and cellular debris. The supernatant was transferred to a 15 mL centrifuge tube for exosome extraction, following the protocol provided with the Exosome Concentration Kit (Rengen Biosciences, Liaoning, China). The extracted exosomes were subsequently characterized using transmission electron microscopy. A total of 20 μL of exosomes were adsorbed onto TEM 200 copper mesh at room temperature for a duration of 10 min. Subsequently, the exosomes were stained with phosphotungstic acid for a period of 1 min. After undergoing a 30-min drying process using an infrared lamp, the copper mesh was carefully positioned inside a sample box, and electron microscopic images were captured at an acceleration voltage of 80 kV.

### Western blot assay

The Cell proteins were extracted using RIPA buffer (50 mM Tris–Cl (pH 7.5), 150 mM NaCl, 0.2 mM EDTA and 2% NP-40) and quantified using a BCA protein assay kit (Beyotime Biotechnology). A total of 20 μg of protein was separated on 10% SDS-PAGE gels and subsequently transferred to 0.22 μm PVDF membranes (Millipore). The membranes were blocked using 5% skim milk powder and subsequently incubated with anti-PEDV N protein antibodies (Medgene Labs) at 4 °C overnight. The membranes were then incubated with the corresponding secondary antibodies, and an enhanced chemiluminescence (ECL) detection system (Bio-Rad) was used to detect the protein bands. HSP90 (R1510-29, HuaBio) were used as the loading control for the Western blot assay.

### Luciferase reporter assay

The co-transfection of the ssc-miR-1343 mimics, wild-type and mutant plasmids, or control plasmids was performed in 293T cells. After a 48-h incubation period, the luciferase activity was measured using the dual-luciferase reporter gene assay kit (Beyotime Biotechnology). Then, the ratio of firefly to Renilla luciferase activity was calculated.

### RNA immunoprecipitation assay

The RNA immunoprecipitation (RIP) assay was performed using the RNA Immunoprecipitation Kit (Geneseed Biotech) in order to investigate the binding interaction between ssc-miR-1343 and Ago2 proteins. For the RIP assay, 5 μg of Ago2 antibody (Abcam) and 5 μg of IgG antibody (Dia-An Biotech) were utilized. The RNAs that were co-precipitated were eluted and subsequently detected using reverse transcription (RT)-PCR.

### Statistical analysis

Data processing was performed using Excel software, and the data are presented as the mean ± SD. SPSS software was used for statistical analysis. A two-sided Student’s t-test was used to analyze the differences between the two groups. A standard analysis of variance (ANOVA) was used to analyze the differences in more than two groups. In all analyses, ^*^P < 0.05; ^**^P < 0.01; ^***^P < 0.001 for the comparison of the indicated treatments.

## Results

### PEDV infection alters miRNA profiles in intestine between PEDV-infected and healthy piglets

**Figure 1:**
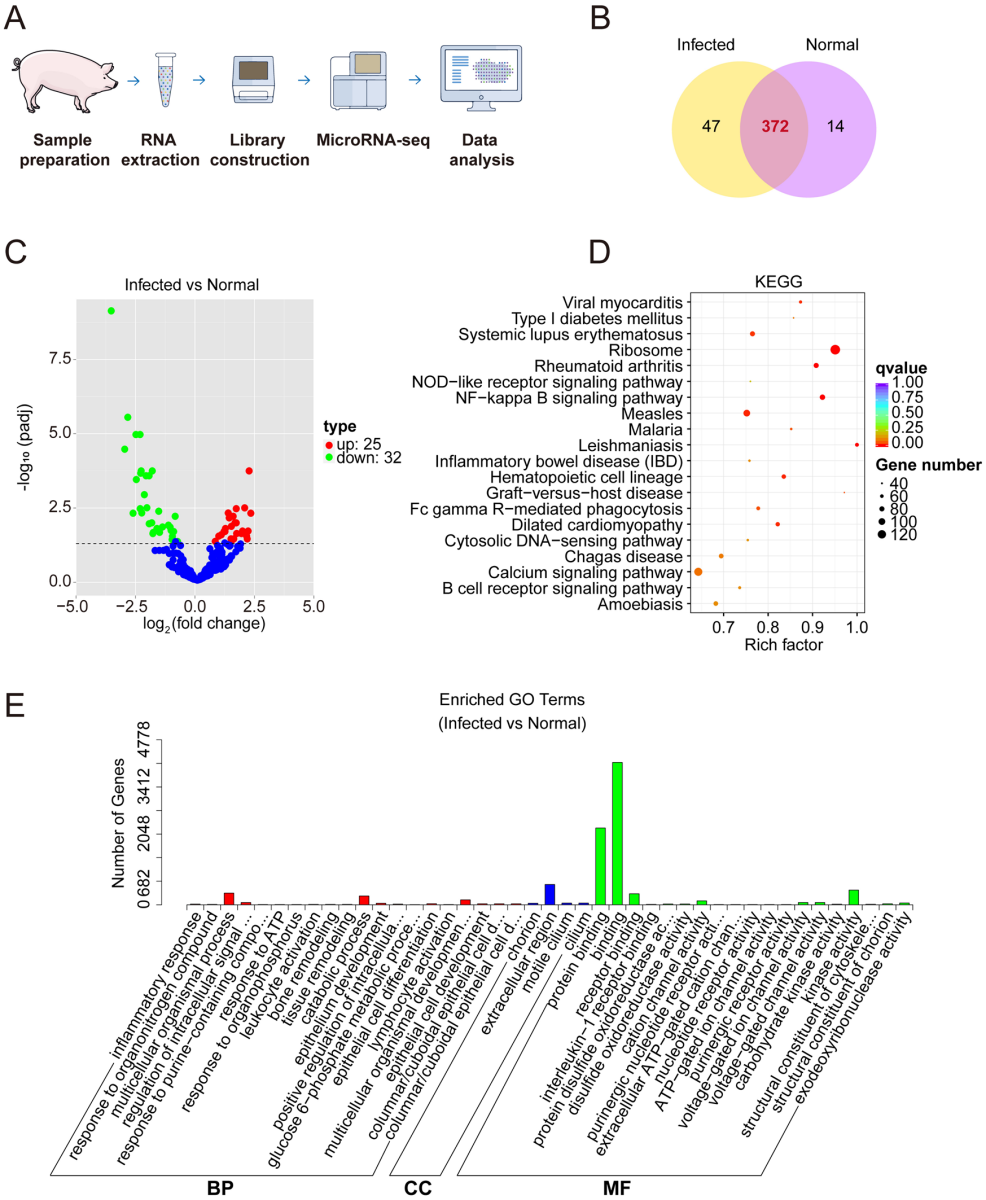
**The characteristics of the miRNA expression profile of intestinal mucosa infected with PEDV.** **A** Workflow of miRNA-seq (*n* = 4). **B** Overlap of miRNA numbers between PEDV-infected and normal groups. **C** Volcano of DE miRNAs. Horizontal coordinates represent the fold change in miRNA expression in different groups, blue dots indicate non-significantly different miRNAs, red dots indicate significantly up-regulated differential miRNAs, and green dots indicate significantly down-regulated differential miRNAs. **D** Bubble chart of KEGG enrichment of candidate target genes. The vertical axis indicates the pathway name, the horizontal axis indicates the Rich factor, the size of the dots indicates the number of candidate target genes in the pathway, and the color of the dots corresponds to the different Qvalue ranges. **E** GO enrichment of candidate target genes. Three different classifications represent the three basic classifications of the GO term (from left to right, biological process, cellular component, and molecular function).

Our previous study performed lncRNA-seq and RNA-seq analyses on PEDV-infected and healthy piglets in order to identify the non-coding RNAs (ncRNAs) that are involved in the process of PEDV infection [17]. MiRNAs, as a type of ncRNAs, were investigated in this study to explore their involvement in pigs' resistance against PEDV infection. The same samples were used to conduct the miRNA-seq, as shown in Figure 1A. A total of 372 miRNAs exhibited an overlap between PEDV-infected and normal piglets (Figure 1B). Following PEDV infection, a total of 25 DE miRNAs were found to be up-regulated; meanwhile, 32 DE miRNAs were down-regulated (Figure 1C). RNAhybrid and miRanda were employed to identify the potential target genes of miRNAs. Subsequently, enrichment analysis revealed that these target genes were significantly enriched in the ribosome pathway and the binding GO term (Figures 1D and E).

### Analysis of competitive endogenous RNA (ceRNA) regulatory networks in the PEDV infection process

**Figure 2:**
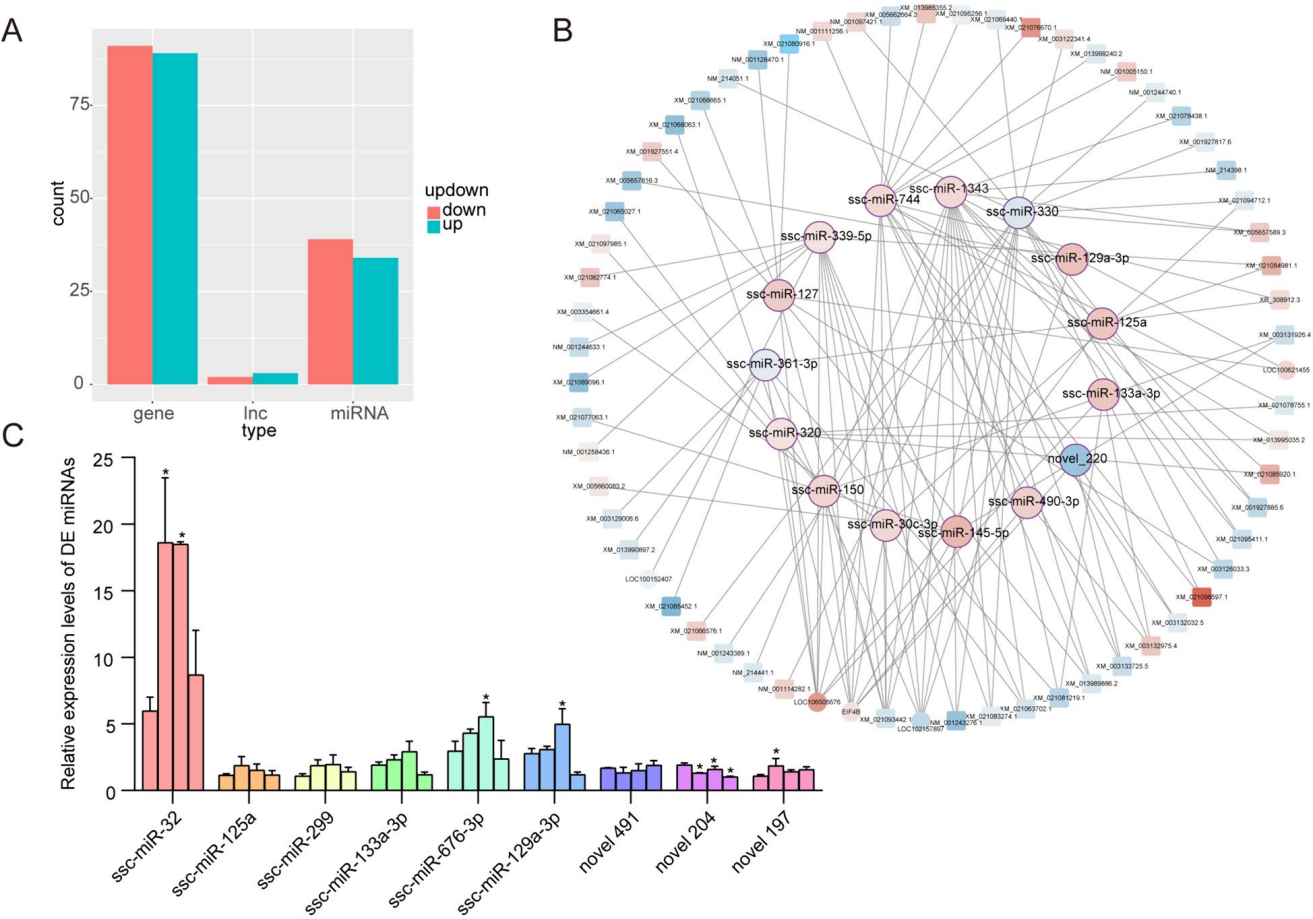
**The construction of the lncRNA-miRNA-mRNA network.** **A** A Histogram of miRNAs, target lncRNAs, and target mRNAs. The miRNAs are DE miRNAs identified through miRNA-seq. Target lncRNAs are DE lncRNAs predicted to bind miRNAs from lncRNA-seq [17]. Target mRNAs are DE mRNAs predicted to bind miRNAs from RNA-seq [17]. **B** The interactions network of ceRNAs. **C** Expression changes in differentially expressed miRNAs at different time points in PEDV-infected IPEC-J2 cells (*n* = 3). The four columns of the same color from left to right represent 0, 12, 24, and 48 hpi, each showing differences compared to 0 h, ^*^P < 0.05.

CeRNAs (most commonly lncRNAs and circRNAs) can affect the microRNA-induced gene silencing through binding to microRNAs via the microRNA response elements (MREs), which represent a new mode of gene expression regulation [18]. To construct these ceRNA networks, we utilized the lncRNA-seq and RNA-seq data obtained from our group’s previous study [17]; then, we predicted the miRNA targeting lncRNA and mRNA using miRanda. lncRNA, as a type of ceRNA, can bind miRNA competitively with mRNA [19]. The findings of this study indicate that in pigs, the number of mRNAs bound with miRNAs far exceeded the number of lncRNAs bound to them (Figure 2A). Additionally, lncRNA–miRNA–mRNA pairs were constructed, and a total of 15 DE miRNAs were identified from the networks (Figure 2B). The expression changes in nine DE miRNAs in IPEC-J2 cells during different time points of PEDV infection were randomly verified. The findings revealed that ssc-miR-32, ssc-miR-676-3p, ssc-miR-129 m-3p, and novel197 exhibited a significant upward trend before their expression decreased; meanwhile, novel204 demonstrated a significant downward trend (*P* < 0.05) (Figure 2C).

### Exosomal ssc-miR-1343 inhibits PEDV infection

**Figure 3:**
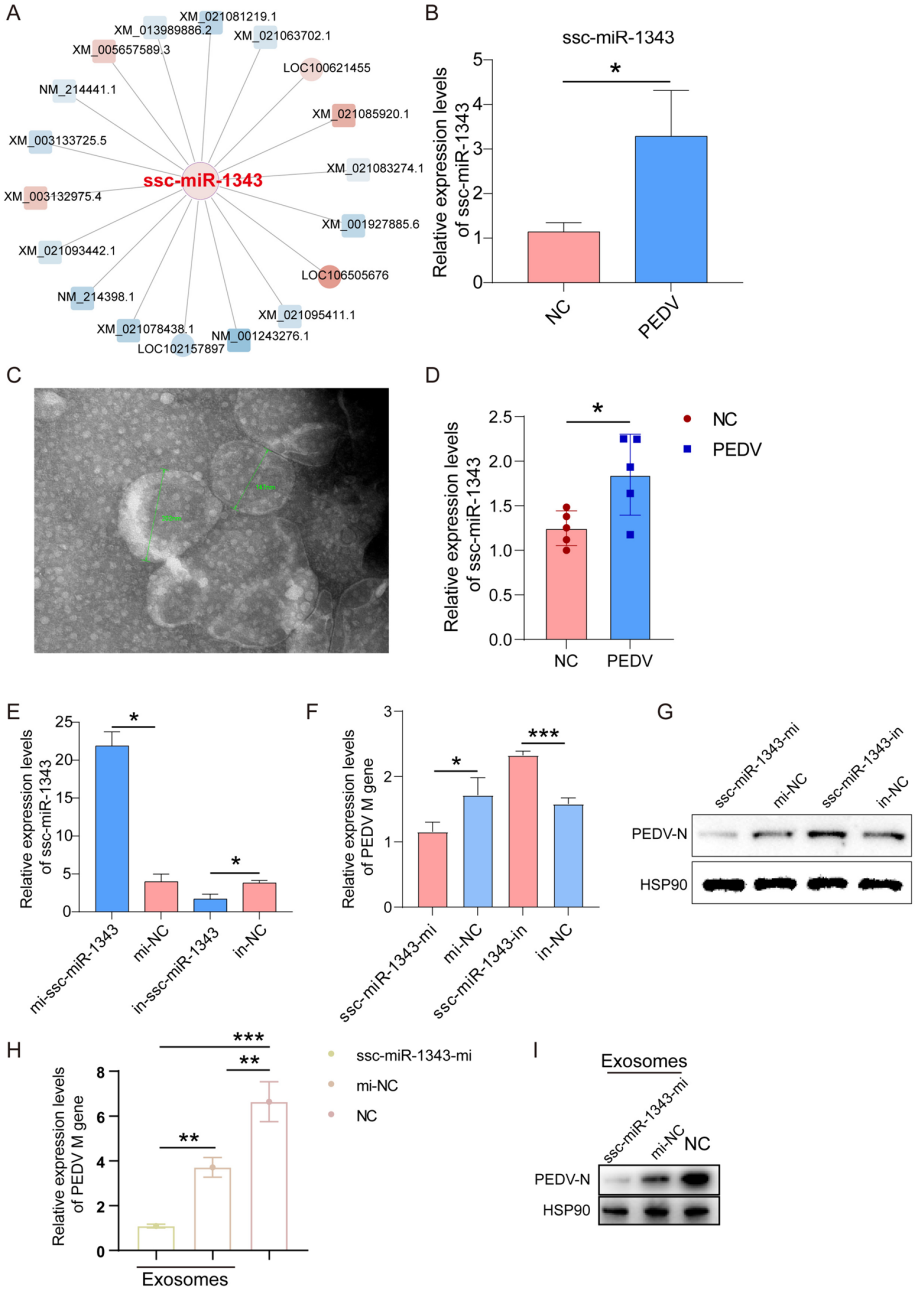
**The ssc-miR-1343 in exosomes can effectively inhibit PEDV replication as a potential functional miRNA.** **A** The predicted target genes and lncRNAs of ssc-miR-1343. **B** The expression of ssc-miR-1343 in PEDV-infected and control IPEC-J2 cells (*n* = 3). **C** Transmission electron microscopy identification of extracted exosomes. The green line segment represents the diameter of exosomes. **D** The expression of ssc-miR-1343 in exosomes of PEDV-infected and control groups (*n* = 5). **E** qRT-PCR was performed to detect the efficiency of ssc-miR-1343 overexpression and interference (*n* = 3). **F** qRT-PCR was performed to detect the effect of ssc-miR-1343 overexpression and interference on mRNA expression of PEDV *M* gene. **G** Western blot assays were performed to detect the effect of ssc-miR-1343 overexpression and interference on PEDV N protein expression. **H** and **I** qRT-PCR and Western blotting to detect whether exosomes deliver ssc-miR-1343 to inhibit viral replication during PEDV infection. We collected the exosomes from mimics-treated IPEC-J2 cells for 24 h. IPEC-J2 cells were incubated at a concentration of 20 μg/mL exosomes for 24 h and then infected with PEDV. Total RNA and proteins were collected 24 hpi after infection for qRT-PCR and Western blot, respectively.

We analyzed the ceRNA networks and found that ssc-miR-1343 targeted18 genes, representing the highest number amongst the results of the analyses (Figure 3A). The qRT-PCR results showed that the expression level of ssc-miR-1343 increased significantly after 48 h of PEDV infection in IPEC-J2 cells (*P* < 0.05) (Figure 3B). A recent study showed that miRNAs can be carried and transported by exosomes, thereby demonstrating that exosomes have a role to play [20]. Therefore, we isolated and extracted the exosomes from IPEC-J2 cells (Figure 3C). The qRT-PCR results demonstrated that ssc-miR-1343 was localized in the exosomes, and its expression significantly increased after PEDV infection (*P* < 0.05) (Figure 3D). The mimics and the inhibitor were used for the overexpression and knockdown of ssc-miR-1343, respectively. The expression of ssc-miR-1343 could be efficiently mimicked and effectively inhibited by the mimics and inhibitor (Figure 3E). The subsequent detection revealed that treatment with ssc-miR-1343 mimics decreased PEDV *M* mRNA and N protein expression, while treatment with the inhibitor up-regulated *M* mRNA and N protein expression (Figures 3F and G). This suggests that ssc-miR-1343 may have potential as an antiviral molecule in host cells during PEDV infection. We further verified whether ssc-miR-1343 is secreted extracellularly via exosomes and then travelled to paracellular cells to exert its function. The qRT-PCR and Western blot results showed that exosome treatment significantly inhibited PEDV replication (*P* < 0.01), and mimics-treated exosomes further significantly inhibited PEDV replication (*P* < 0.01) (Figures 3H and I), indicating that ssc-miR-1343 could deliver by exosomes and thus inhibit PEDV infection.

### ssc-miR-1343 binds to the 3′ UTR region of *FAM131C* and regulates its mRNA stability

**Figure 4:**
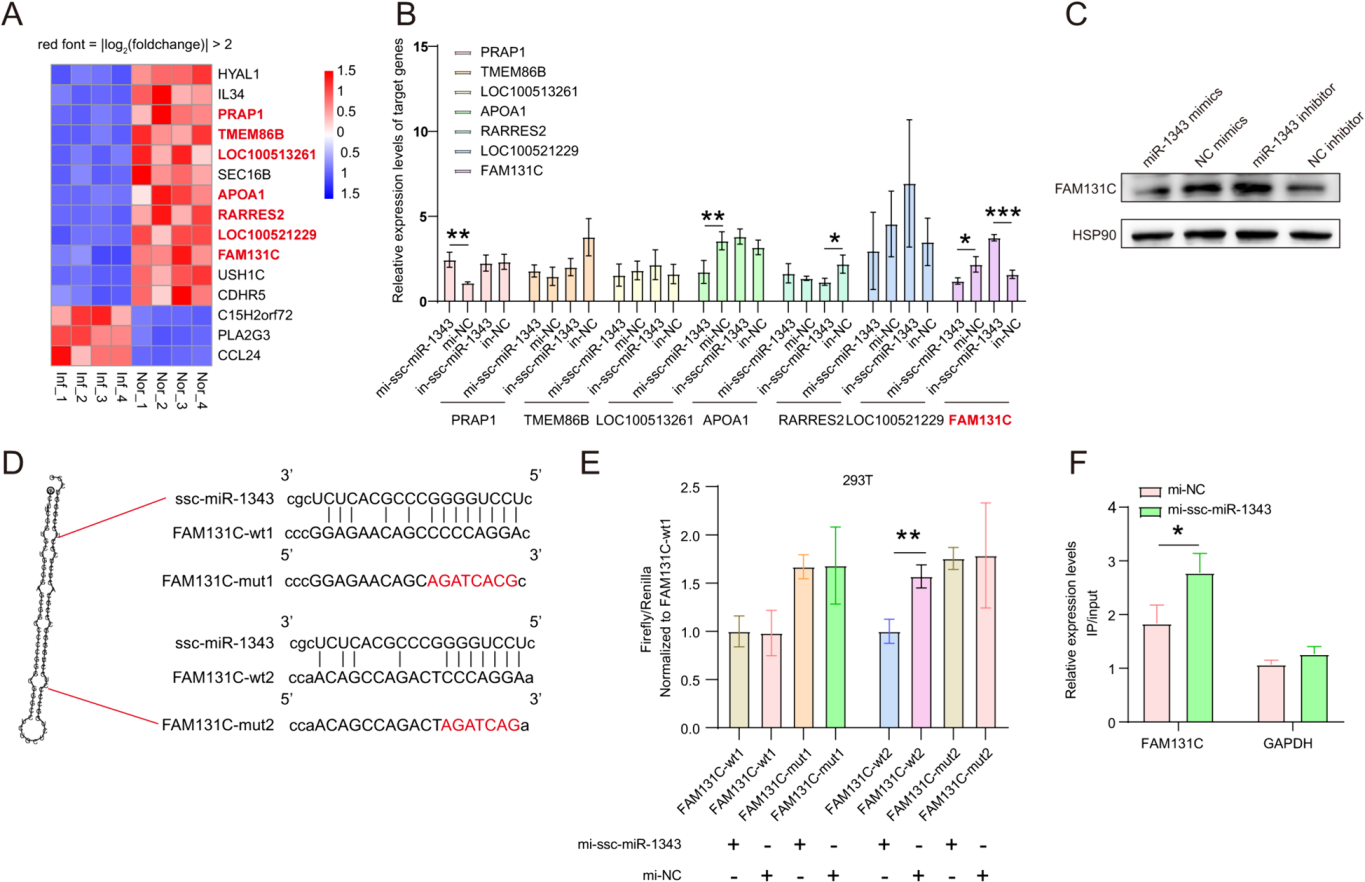
**The screening and identification of target genes of ssc-miR-1343.** **A** A heatmap illustrating the predicted target genes of ssc-miR-1343. Red genes are defined as |log(foldchange)|> 2. **B** The effect of mimics and inhibitor treatment of ssc-miR-1343 on the mRNA expression of the predicted target genes. **C** The effect of mimics and inhibitor treatment of ssc-miR-1343 on the expression of FAM131C protein. **D** The effect of mimics and inhibitor treatment on the binding sites of ssc-miR-1343 and FAM131C. Red bases represent mutated bases. **E** Effect of mimic treatment of ssc-miR-1343 on FAM131C wild-type and mutant dual luciferase activity. **F** The effect of mimic treatment of ssc-miR-1343 on the binding capacity of FAM131C to Ago2.

We set a threshold value of |log_2_ fold change values|> 2, and identified seven target genes of ssc-miR-1343, namely *PRAP1*, *TMEM86B*, *LOC100513261*, *APOA1*, *RARRES2*, *LOC100521229*, and *FAM131C* (Figure 4A). The qRT-PCR results showed that only *FAM131C* was down-regulated after treatment with the ssc-miR-1343 mimics and up-regulated after treatment with the inhibitor (Figure 4B). In addition, its protein level exhibited consistent changes (Figure 4C). Therefore, it is hypothesized that *FAM131C* is a target gene of ssc-miR-1343. Mammalian miRNAs primarily target mRNAs via binding to the cis-regulatory sites in the 3′ UTRs, leading to posttranscriptional repression [21]. An important target motif has been identified as a ~ 7-nt site that matches the seed region of the miRNA [22]. To further validate the mechanism of the ssc-miR-1343-targeted regulation of *FAM131C*, we constructed both mutant and wild-type vectors for the two predicted ssc-miR-1343 binding sites. We then co-transfected these vectors with ssc-miR-1343 mimics into 293T cells (Figure 4D). Using a dual-luciferase assay, we found that only the dual luciferase activity was significantly inhibited by the mimics after the *FAM131C*-wt2 mutation; meanwhile, the *FAM131C*-wt1 mutation remained unchanged (Figure 4E). This indicates that ssc-miR-1343 affects the mRNA stability of *FAM131C* via binding to the region of *FAM131C*-wt2. Argonaute-2 (Ago2) is one of the key participants in the canonical biogenesis of miRNA [23]. The RIP-qPCR results showed that *FAM131C* mRNA enrichment was significantly increased in the Ago2-immunoprecipitate of the mimic treatment groups (Figure 4F). This confirms that ssc-miR-1343 promotes the binding of the Ago2 protein to *FAM131C* mRNA.

### *FAM131C* inhibits host’s innate immune response to facilitate PEDV replication

**Figure 5:**
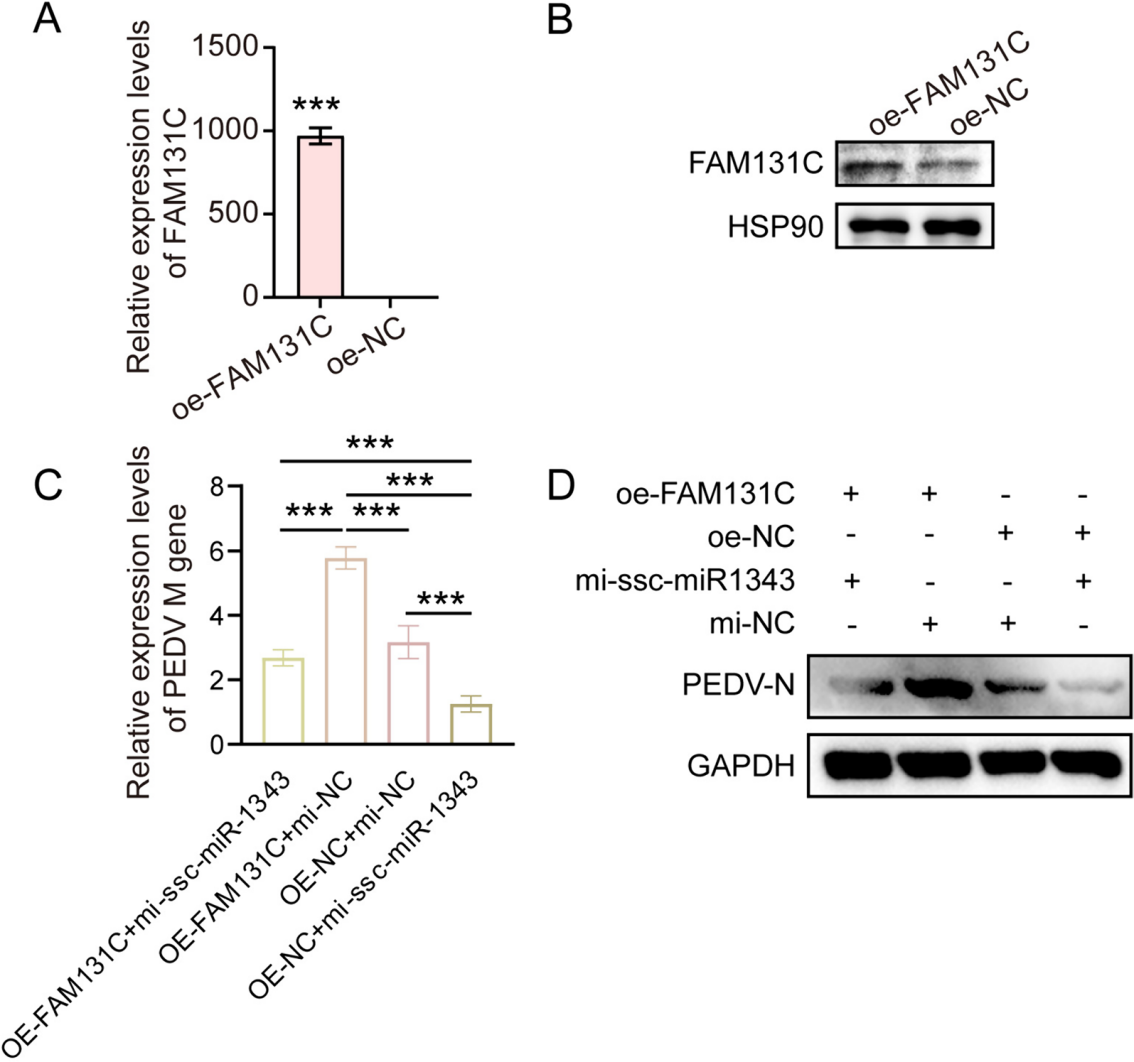
**ssc-miR-1343 inhibits PEDV N protein expression induced by FAM131C overexpression.** **A** qRT-PCR to detect the mRNA expression efficiency of FAM131C overexpression. **B** Western blot analysis was performed to detect the protein expression efficiency of FAM131C overexpression. **C** The impact of ssc-miR-1343 and FAM131C overexpression treatment on the mRNA expression of PEDV *M* gene. **D** The impact of ssc-miR-1343 and FAM131C overexpression treatment on the expression of PEDV N protein.

**Figure 6:**
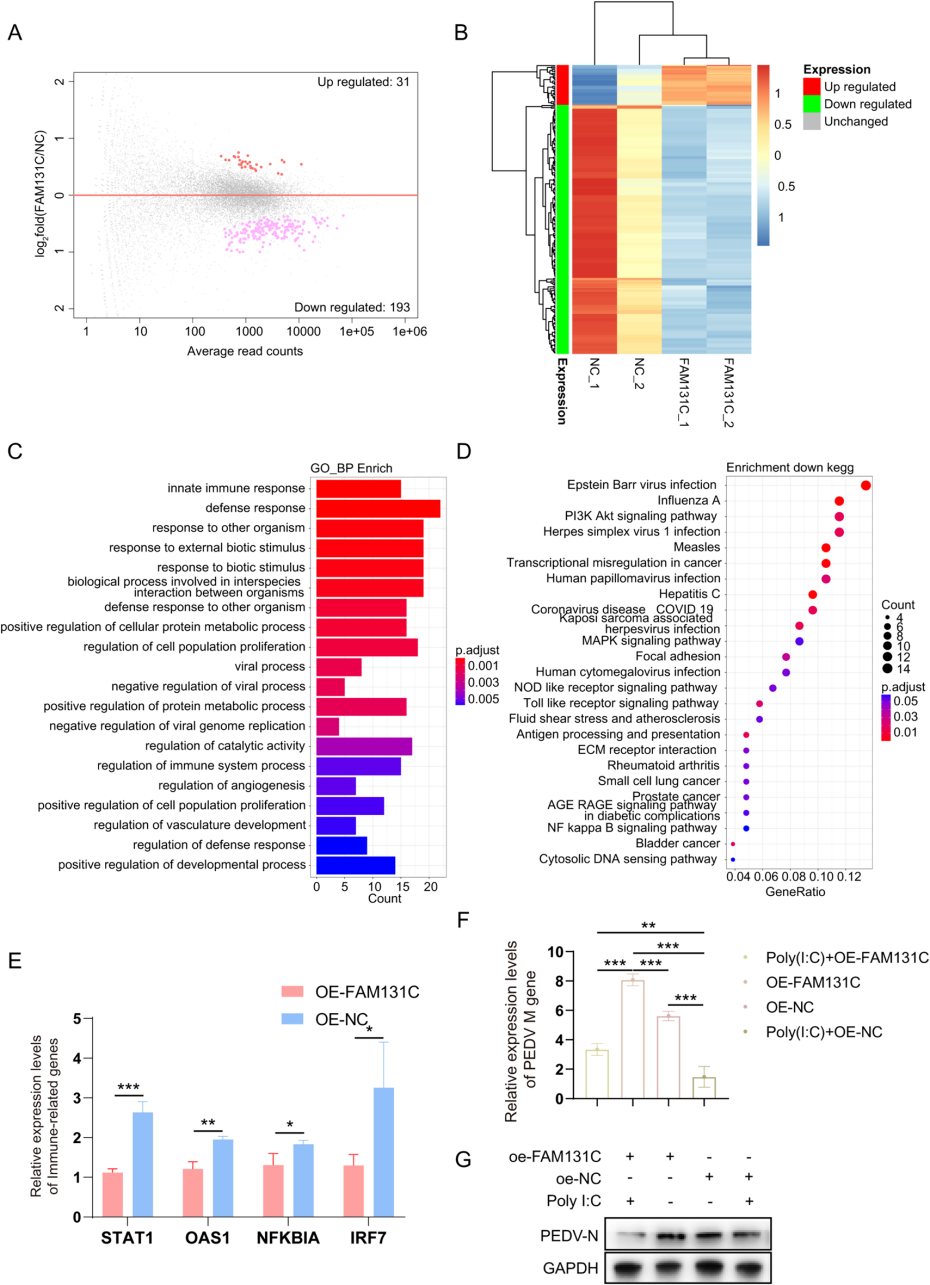
**Effect of FAM131C overexpression on transcription in IPEC-J2 cells.** **A** M-versus-A plot (MA) of DEGs between the FAM131C overexpression group and the NC group. **B** A heatmap of gene expression across various groups. **C** GO BP enrichment analysis of DEGs between the FAM131C overexpression group and the NC group. **D** The top 20 KEGG pathways of down-regulated DEGs between the FAM131C overexpression and the NC group. **E** The impact of FAM131C overexpression treatment on the mRNA expression of immune-related genes. **F** and **G** qRT-PCR and Western blotting to detect whether FAM131C promotes PEDV replication by inhibiting the innate immune response. We treated FAM131C overexpressing cells with 5 µg/mL Poly (I:C) (MCE) for 24 h, followed by infection with PEDV for 24 hpi. Total RNA and protein were collected for qRT-PCR and Western blot, respectively.

To investigate the potential mechanism of *FAM131C* in PEDV infection, we firstly generated a *FAM131C* overexpressing IPEC-J2 cell line, and we confirmed that this overexpression was efficient at both the mRNA and protein levels (Figures 5A and B). After adding the ssc-miR-1343 mimics to the FAM131C overexpression cell line, we observed that the mimics had the capacity to prevent the increase in PEDV replication that followed the overexpression of FAM131C (Figures 5C and D). We subsequently performed RNA-seq to identify the DEGs following *FAM131C* overexpression. A total of 224 DEGs were screened, with 31 DEGs up-regulated and 193 DEGs down-regulated (Figure 6A). This indicates that *FAM131C* overexpression resulted in a higher number of down-regulated genes in IPEC-J2 cells following PEDV infection (Figure 6B). Notably, the down-regulated DEGs were significantly enriched in immune-related GO terms and pathways (Figures 6C and D). Finally, we randomly selected four immune-related genes (*STAT1*, *OAS1*, *NFKBIA*, and *IRF7*) and examined their expression after overexpressing FAM131C. We found that the expression of all these genes significantly decreased following overexpression of FAM131C (*P* < 0.05), consistent with the results of the RNA-seq (Figure 6E). This suggests that *FAM131C* may suppress the host’s immune response. Poly (I:C) is an agonist of the innate immune response. We found that Poly (I:C) could inhibit PEDV replication facilitated by overexpression of FAM131C. From this, it is presumed that FAM131C could promote PEDV replication by inhibiting the host’s immune response.

## Discussion

Although miRNA expression profiles have been reported to be associated with PEDV infection [8–10], the existing studies have primarily focused on IPEC-J2 or Vero cells rather than those in the porcine intestine. As a result, the roles of miRNAs in PDEV infection have remained unclear. In our study, we identified 25 upregulated miRNAs and 32 downregulated miRNAs. Based on the lncRNA-seq and RNA-seq data from the previous study, we constructed a ceRNA network and identified 15 DE miRNAs. There are a few studies in the literature on covering these DE miRNAs in viral infections. MiR-125b inhibits the hepatitis B virus [24], meanwhile, miR-145-5p is associated with the influenza A virus H3N2 [25]. MiR-127-3p suppresses the oncogenic herpesvirus [26]. MiR-127-3p suppresses oncogenic herpesvirus [23]. MiR-361-3p was downregulated during ZIKV infection [27]. MiR-150 [28], miR-320, miR-744 [29], and miR-339-5p [30] are associated with infection with the porcine reproductive and respiratory syndrome virus (PRRSV) infection. MiRNAs are a class of highly conserved non-coding RNAs. The studies on viral infections in other species that involve these miRNAs suggest that these DE miRNAs may potentially be associated with PEDV infection. In addition, our previous study showed that miR-129a-3p inhibits PEDV replication through the targeting of the EDA-mediated NF-κB pathway in IPEC-J2 cells [14]. It also further corroborates that our miRNA-seq analysis helps to identify potential functional miRNAs during PEDV infection. However, it was puzzling that the comparison of miRNA-seq between this study and others in PEDV infection revealed that only a few DE miRNAs overlapped (Additional file 2). We speculate that this may be partly due to inconsistent thresholds for DE miRNAs. Another larger possibility is that other studies have focused on the cellular level, while this study specifically selected piglet mucosal samples.

Exosomes, which are small vesicles derived from various cell types, are actively secreted into the extracellular space, and play a crucial role in intercellular communication through facilitating the transport of proteins, mRNAs, miRNAs, and nucleic acids between cells [20]. In this study, we analyzed the localization of the potential functional miRNA ssc-miR-1343 in IPEC-J2 cells, and ssc-miR-1343 in exosomes can inhibit PEDV replication. The results showed that ssc-miR-1343 was expressed in exosomes, and its expression level significantly increased after PEDV infection. This finding led us to speculate that ssc-miR-1343 may be transmitted between cells through exosomes and play a functional role. Therefore, we selected ssc-miR-1343 for a follow-up study and discovered that it could effectively inhibit PEDV infection. This finding suggests that ssc-miR-1343 has the potential to be used as an antiviral molecule.

**Figure 7:**
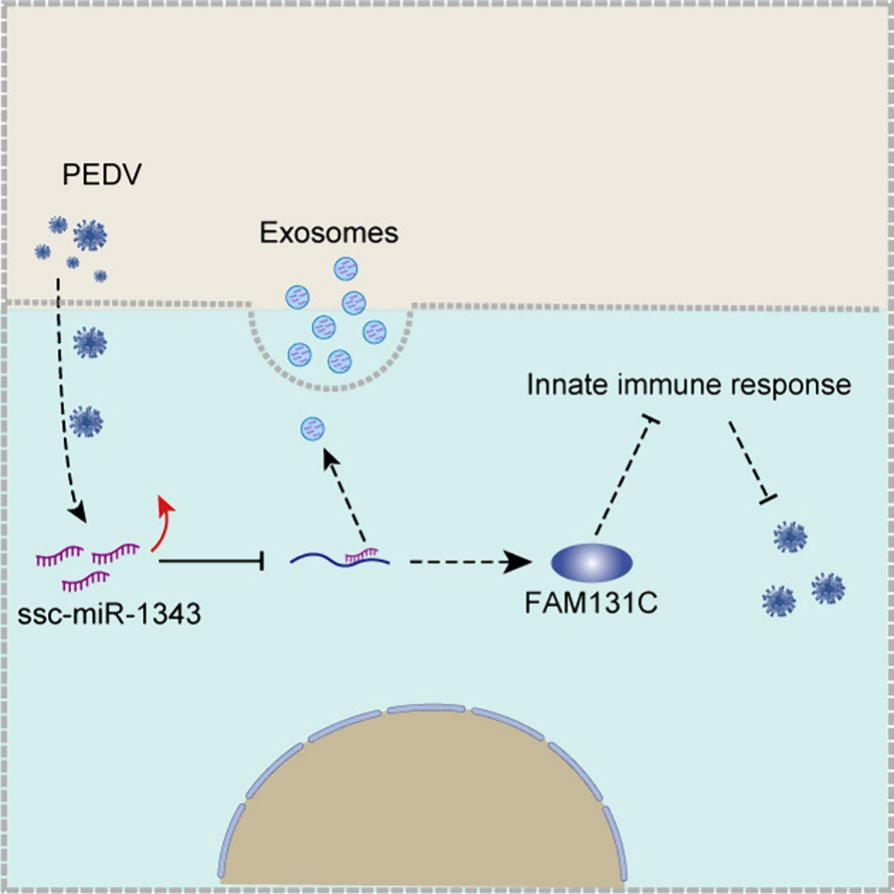
**Mechanistic hypothesis of ssc-miR-1343 regulation of PEDV replication**.

We next explored the target genes of ssc-miR-1343. The current studies have partially identified the targets of miR-1343 in different species. miR-1343-5p modulates IFN-I responses to facilitate the replication of feline panleukopenia virus through direct targeting of the *IRAK1* gene [31]. In colorectal carcinoma, TEAD4 nuclear localization and regulation was demonstrated using miR-4269 and miR-1343-3p [32]. A study also speculated that miR-1343 plays a role in the regulation of inflammation and apoptosis in pigs [33]. Additionally, miR-1343 regulates the pluripotency of porcine stem cells through the repression of OTX2 expression [34]. However, miRNAs are a class of pluripotent non-coding RNAs that target a large number of genes. In this study, we predicted the potential targets of ssc-miR-1343 using pre-transcriptomic data. We then conducted RIP-qPCR and dual luciferase assays to determine that ssc-miR-1343 can bind to the 3'-UTR of *FAM131C* and destabilize its mRNA. The follow-up functional assays also demonstrated that the overexpression of *FAM131C* significantly enhanced the infection efficiency of PEDV by suppressing the immune response. These findings indicate that the inhibitory role played by exosomal ssc-miR-1343 in relation to *FAM131C* expression may be a significant protective factor in PEDV infection in pigs (Figure 7).

However, the function of the *FAM131C* gene remains understudied, although it may be associated with the maintenance of male fertility [35]. Our results suggest that *FAM131C* can effectively promote the replication of PEDV. In this study, RNA-seq was subsequently performed on both the overexpressed *FAM131C* and control groups at 24 h post PEDV infection to investigate the potential mechanism of *FAM131C* in increasing PEDV infection. The results showed that the majority of DEGs were down-regulated and enriched in immune-related GO terms and pathways. This suggests that FAM131C acts as a suppressor of immune gene expression in the porcine intestinal epithelial cells.

The innate immune response of the host is the first line of defense against the viral invasion of small intestinal epithelial cells. During viral infection and the replication processes, the pathogen-associated molecular patterns (PAMPs) are recognized via pattern recognition receptors (PRRs) [36]. After PEDV invades the host cells, its genomic nucleic acids, double-stranded RNA (dsRNA), and replication-produced proteins activate two major signaling pathways in the host innate immune response (the RIG-I receptor signaling pathway and Toll-like receptor signaling pathway), which in turn induce IFN production for innate antiviral functions [37]. In contrast, IFN can induce the expression of thousands of interferon-stimulated genes (ISGs), many of which have antiviral activity [38]. These antiviral factors can act on various RNA viruses in two ways. Firstly, they stimulate the production of antiviral proteins in body cells, which directly trap and kill viruses. Secondly, they utilize the connection between the body's innate and acquired immunity to suppress viruses in vivo. Therefore, this study hypothesized that the host may promote the production of IFN through the ssc-miR-1343/FAM131C axis and thus resist PEDV infection.

Clearly, there are still some obvious shortcomings in this study. For instance, there is a lack of functional analysis for other potential DE miRNAs. Additionally, the mechanism of the FAM131C regulation of the host’s immune response has not been fully demonstrated. These aspects still need to be verified in future research. This study revealed the differential expression profile of DE miRNAs in the jejunal mucosa of piglets after PEDV infection, and also clarified the antiviral mechanism of the exosomal ssc-miR-1343/FAM131C axis during PEDV infection. These findings provide a useful reference and experimental basis for the further study of the function of exosomal miRNAs during PEDV infection. These studies on exosomes as potential therapeutic targets contribute to the development of small-molecule drugs against PEDV infection.

### Supplementary Information

**Additional file 1. Details of primers**.

**Additional file 2. The comparison of miRNA-seq between this study and others in PEDV infection**.

## Data Availability

The data that generated during the current study are available from the corresponding author on reasonable request.
