# Supplementary material for: Exosomal ssc-miR-1343 targets FAM131C to regulate porcine epidemic diarrhea virus infection in pigs
Source: Vet Res. 2024 Jul 22;55:91. doi: 10.1186/s13567-024-01345-3 (PMC11264985; doi:10.1186/s13567-024-01345-3)
Supplement: Supplementary file 1 — Additional file 1. Details of primers. [file 13567_2024_1345_MOESM1_ESM.docx]

**Additional file 1 Details of primers**

| **Primers (for qPCR)** | **Forward (5’-3’)** | **Reverse (5’-3’)** |
| --- | --- | --- |
| *ssc-miR-1343* | CTCCTGGGGCCCGCACTCTCGC | GTGCAGGGTCCGAGGT |
| *ssc-miR-32* | TATTGCACATTACTAAGTTGC | GTGCAGGGTCCGAGGT |
| *ssc-miR-125a* | TCCCTGAGACCCTTTAACCTGTG | GTGCAGGGTCCGAGGT |
| *ssc-miR-299* | ATGGTTTACCGTCCCACATAC | GTGCAGGGTCCGAGGT |
| *ssc-miR-133a-3p* | TTGGTCCCCTTCAACCAGCTG | GTGCAGGGTCCGAGGT |
| *ssc-miR-676-3p* | CCGTCCTAAGGTTGTTGAGTT | GTGCAGGGTCCGAGGT |
| *ssc-miR-129a-3p* | AAGCCCTTACCCCAAAAAGCAT | GTGCAGGGTCCGAGGT |
| *novel 491* | TCTGTCATTTCTGTAGGC | GTGCAGGGTCCGAGGT |
| *novel 204* | TGAACGGCGCCTGTGTGGTTAGA | GTGCAGGGTCCGAGGT |
| *novel 197* | AAAGATACTAGTAGAGTTCGTTACT | GTGCAGGGTCCGAGGT |
| *PEDV-M* | AGGTCTGCATTCCAGTGCTT | GGACATAGAAAGCCCAACCA |
| *PRAP1* | CATCTGGACCCTGGGTTGTG | GATCTTGACAAGGGCCTGGG |
| *TMEM86B* | GTTCTCAGGGGTCACAGGC | CACGTGCAGCTGTTGAGC |
| *LOC100513261* | GGAGGAAGGGTAAGGGACCA | TTCAATCCAGCCGACGGAAA |
| *APOA1* | TTCTGGCAGCAAGATGACCC | TGCCACTGTCTTTGATCGCA |
| *RARRES2* | TCAAGCCCAACGGGAGAAAG | TAGTAGCTGTGGGGGTCCTC |
| *LOC100521229* | CTTTGATCAAGCTGCCACCTC | GGGGCTGAGGCTCCTTATTG |
| *FAM131C* | ATGGAGTGAGTCGGCAGGAT | CTTTGCACCACTCCCACAAG |
| *STAT1* | CAAAGGAAGCCCCAGAACCT | ACTTGTTCCATGTCACGTCAA |
| *OAS1* | CCGAGATGGATACCCCTGTTAG | CGAGGAGCCACCCTTCACA |
| *NFKBIA* | AACCAGCCAGAAATCGCTGA | TGCAGACATGTGTGGCCATT |
| *IRF7* | AAAACCAACTTCCGCTGTGC | TTGAAGCCTGGGCCTTCTCC |
| *GAPDH* | ACATCATCCCTGCTTCTACTGG | CTCGGACGCCTGCTTCAC |
| *U6* | GCTTCGGCAGCACATATACTAAAAT | CGCTTCACGAATTTGCGTGTCAT |
